# Isolated melanoma metastasis in a patient with large congenital nevus without detectable primary melanoma: a case report and review of literature

**DOI:** 10.3389/fmed.2024.1427982

**Published:** 2024-10-15

**Authors:** Marta Pabianek, Ilona Jatczak-Grochala, Aleksandra Lesiak, Joanna Narbutt, Aleksandra Siekierko, Olga Stasikowska-Kanicka, Magdalena Ciążyńska

**Affiliations:** ^1^Chemotherapy Unit and One-Day Chemotherapy Unit, Specialist Oncology Hospital NU-MED, Tomaszów Mazowiecki, Poland; ^2^Department of Proliferative Diseases, Nicolaus Copernicus Multidisciplinary Centre for Oncology and Traumatology, Łódź, Poland; ^3^Department of Dermatology, Paediatric Dermatology and Oncology Clinic, Medical University of Lodz, Łódź, Poland; ^4^Laboratory of Autoinflammatory, Genetic and Rare Skin Disorders Medical University of Łódź, Łódź, Poland; ^5^Department of Diagnostic Techniques in Pathomorphology, Medical University of Lodz, Łódź, Poland

**Keywords:** melanoma of unknown primary origin, giant congenital melanocytic nevus, adjuvant treatment, melanoma, primary melanoma

## Abstract

Giant congenital pigmented nevi constitute an extremely diverse group of skin lesions with varying morphologies. These nevi are often associated with many clinical implications, such as increased risk of melanoma and the presence of neurocutaneous melanosis, with melanoma being the primary concern. We present a rare case of a 62-year-old patient with a giant congenital birthmark who reported to the oncology department due to a tumor in the lower abdomen detected during an ultrasound examination. A biopsy of the lesion showed the presence of melanoma metastasis. Four independent dermatologists performed a dermoscopic examination of the patient’s skin and mucous membranes. In the PET/CT examination, apart from the previously described change in the lower abdomen, no metabolically active foci with features of malignant growth were found. The patient underwent surgical removal of the lesion in the lower abdomen. The postoperative histopathological examination confirmed the presence of metastasis of melanoma in the subcutaneous tissue of the abdomen with no connection to the epidermis. The *BRAFV600* mutation was not found in the molecular test. For stage IV R0 melanoma with distant metastasis, with stage T0N0M1a, the only adjuvant treatment option following radical resection is nivolumab. After a rheumatological consultation, the patient was qualified for adjuvant treatment with nivolumab.

## 1 Introduction

Melanoma is one of the most aggressive skin neoplasms, responsible for approximately three-quarters of all skin cancer-related deaths. The incidence of melanoma has steadily increased over the past two decades in nearly all countries in Europe and the United States (1–4). Recently, the treatment of metastatic melanoma has undergone a revolution with the introduction of adjuvant systemic therapy. In recent years, novel drugs for adjuvant systemic therapy for patients who were diagnosed with stage IIB, IIC, III, and IV R0 melanoma were approved by the U.S. Food and Drug Administration (FDA) and the European Medicines Agency (EMA), which improved the prognosis of patients (5–7).

In the absence of a skin lesion, melanoma represents a diagnostic challenge that can delay therapeutic management. Melanoma of an unknown primary origin (MUP) accounts for up to 3% of all melanomas (8) with histologically confirmed melanoma metastasis in lymph nodes, subcutaneous tissue, or visceral sites. The diagnosis of melanoma of unknown primary origin is definitive in the absence of primary cutaneous melanoma, mucous melanoma, or ocular after a thorough physical examination and histological confirmation of previously resected melanocytic lesions. Management of patients with melanoma of unknown primary origin is the same as treatment of patients with metastatic cutaneous melanoma.

Congenital melanocytic nevi are recognized risk factors for melanoma, directly proportional to their number (9). A giant congenital melanocytic nevus, defined as a melanocytic lesion present at birth and reaching 20 cm or more in diameter by adulthood, is associated with a 6% lifetime risk of developing melanoma at the site of the lesion (10, 11).

We report a case of subcutaneous melanoma metastasis in a patient with a giant congenital nevus in the absence of a primary tumor. Our objectives are to discuss the frequency, pathophysiological mechanism, and prognosis of this type of melanoma in relation to the literature.

## 2 Case presentation

A 62-year-old female patient with phototype I (Fitzpatrick scale) skin, presenting with giant congenital melanocytic nevus on her legs and trunk, was admitted to the oncology department after a control ultrasonography examination revealed a tumor in the subcutaneous tissue of the lower abdomen. There were no other systemic complaints, and family history was unremarkable. The patient had been treated for rheumatoid arthritis (RA) with constant doses of methotrexate for 5 years and was under the constant care of a dermatologist and rheumatologist. Abdominal and pelvic ultrasonography revealed a pathological mass of 1 × 1 × 1.3 cm in the subcutaneous tissue. A core needle biopsy of the lesion was performed, which revealed invasive neoplastic cells confirming a subcutaneous melanoma metastasis. Immunohistochemistry showed diffuse cytoplasmic positivity for HMB-45 (anti-gp100) and Melan-A indicating melanocytic malignant lesion.

**Figure 1:**
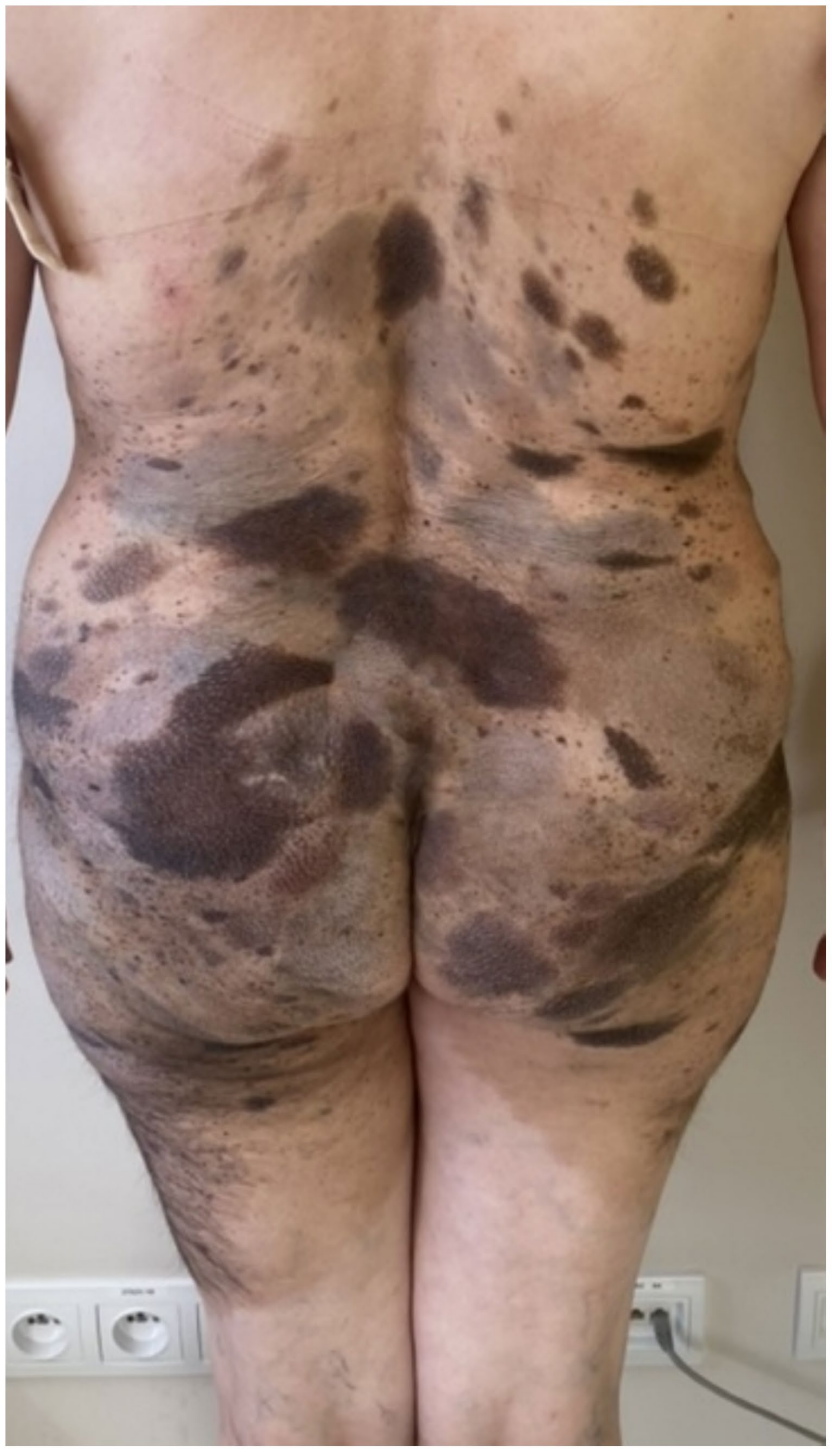
Patient before adjuvant treatment, back view presenting giant CMN with satellite lesions.

**Figure 2:**
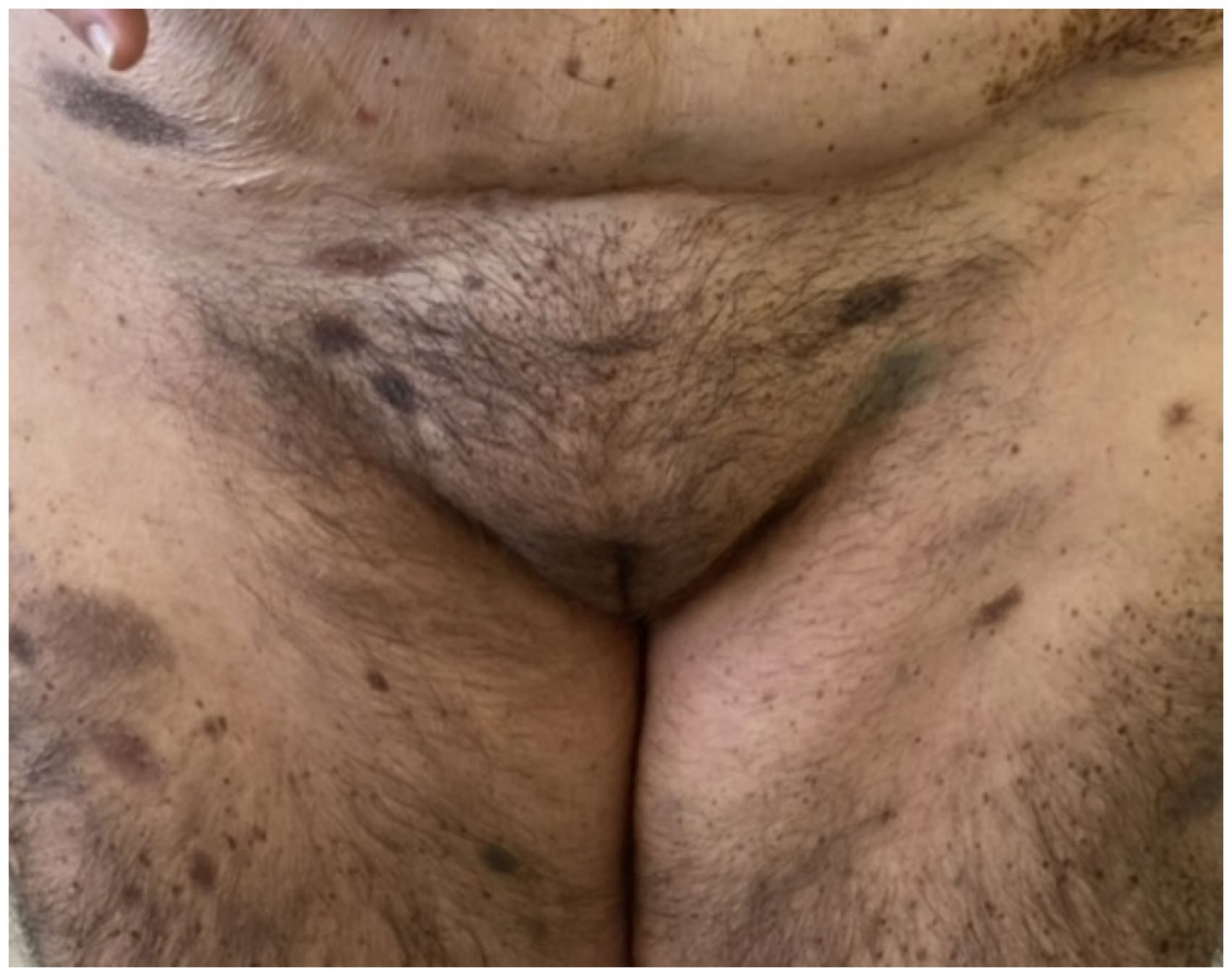
Patient before adjuvant treatment, front view. In the lower abdomen, there is a visible scar after tumor resection within the subcutaneous tissue.

**Figure 3:**
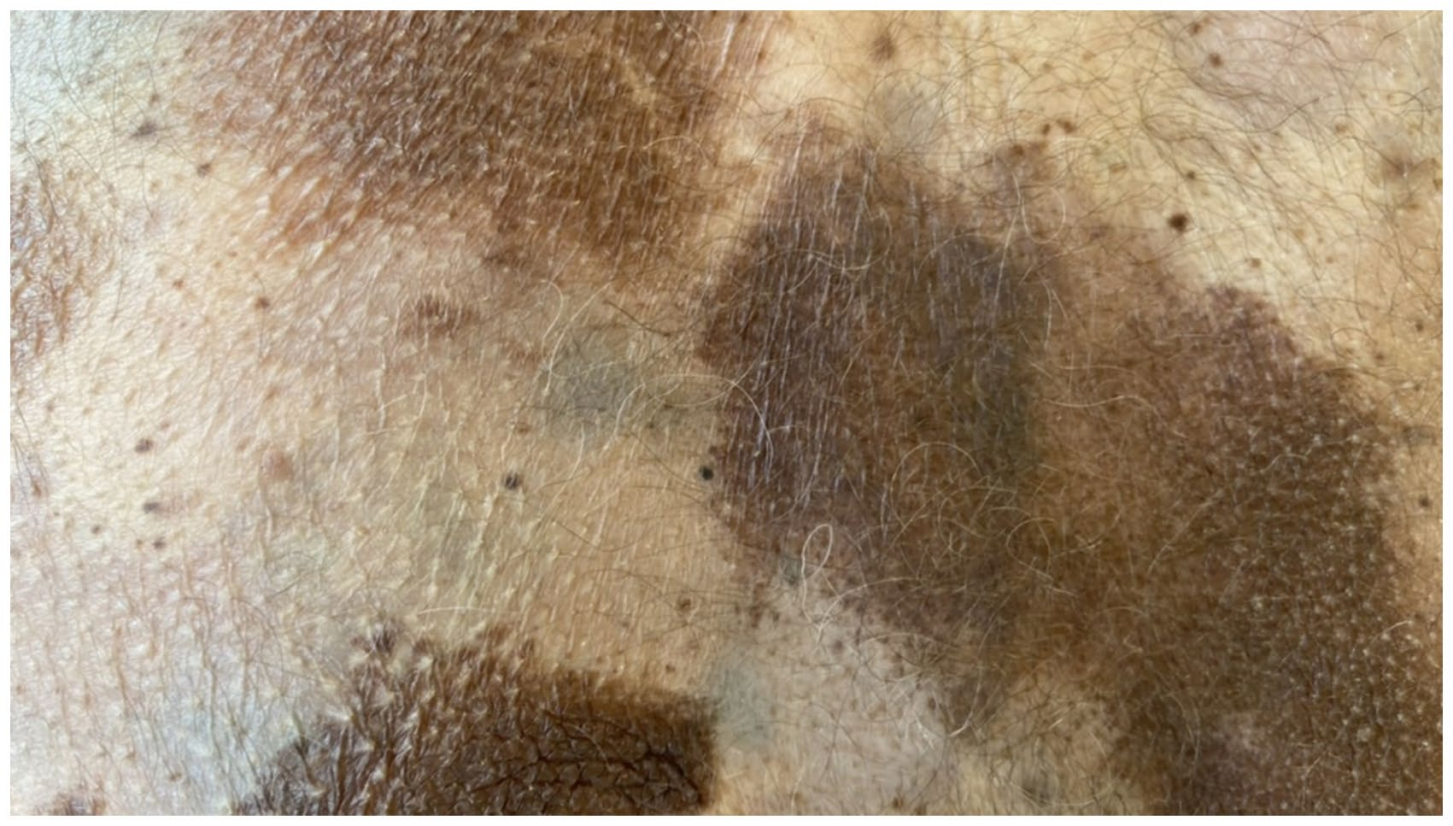
Numerous isolated pigment masses on the patient’s body and limbs.

**Figure 4:**
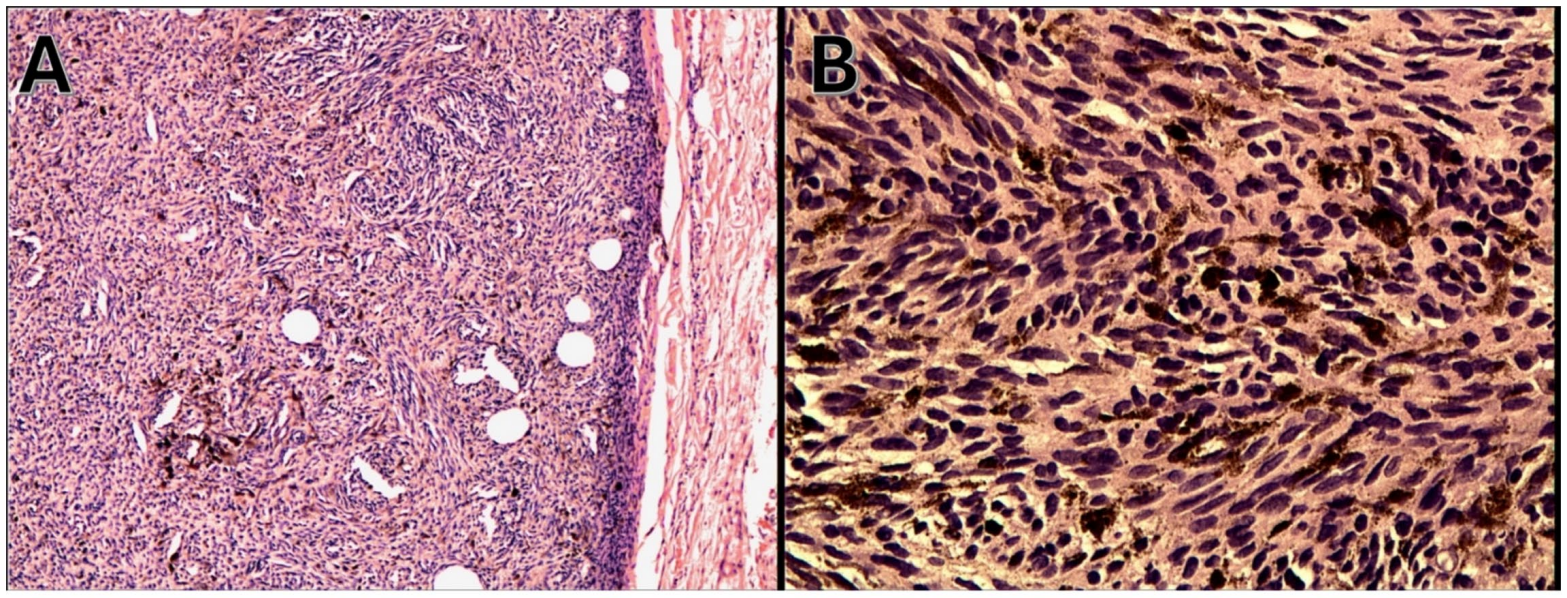
Malignant melanocytes with nuclear atypia and dense melanin pigmentation (hematoxylin and eosin staining), **(A)** magnification 100x and **(B)** 200x.

**Figure 5:**
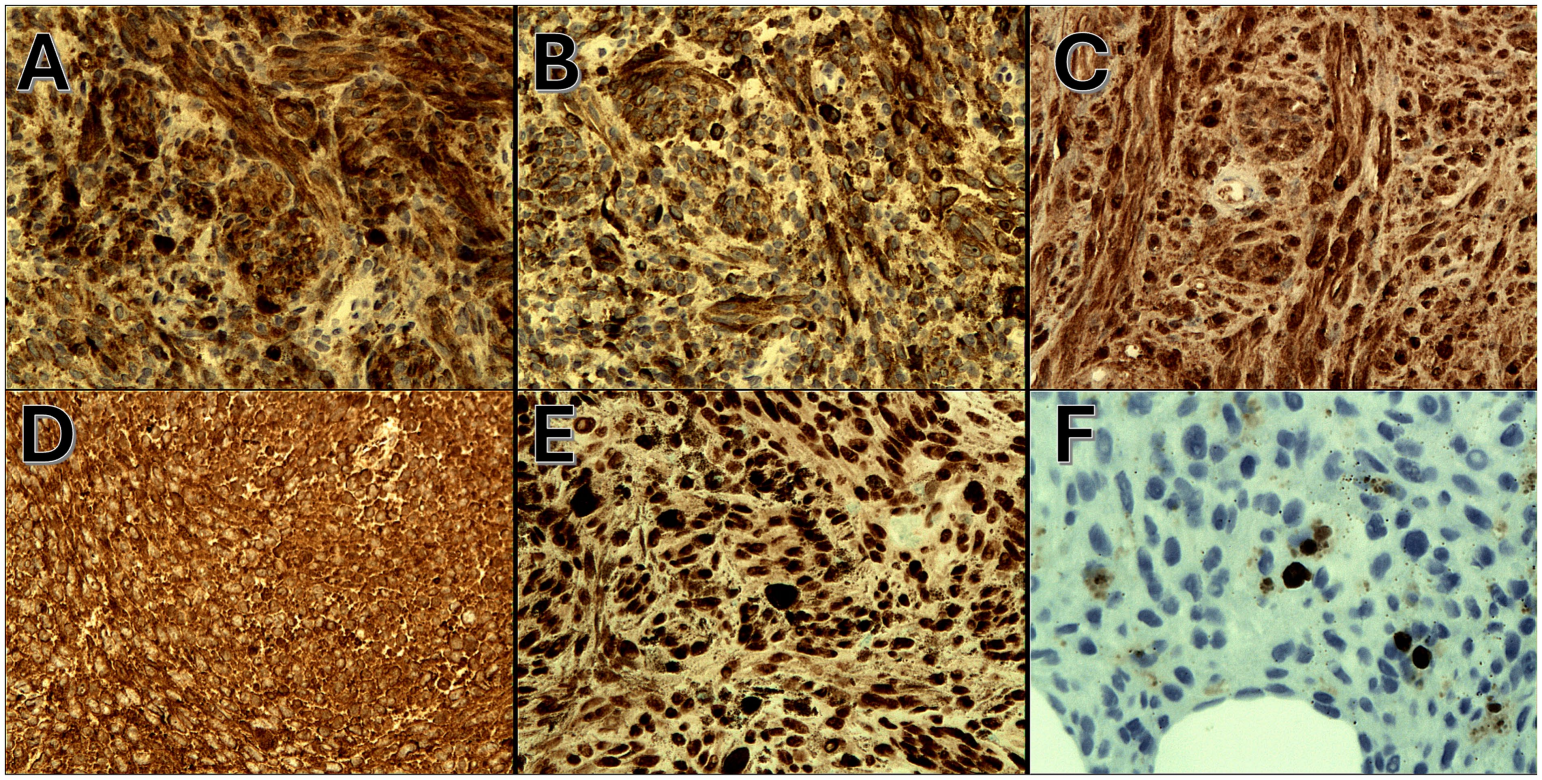
**(A)** Positive cytoplasmic HMB-45 immunoexpression (IHC, magnification 200x), **(B)** positive cytoplasmic Melan-A immunoexpression (IHC, magnification 200x), **(C)** positive cytoplasmic s-100 immunoexpression (IHC, magnification 200x), **(D)** positive cytoplasmic Vimentin immunoexpression (IHC, magnification 200x), **(E)** positive nuclear SOX-10 immunoexpression (IHC, magnification 200x), and **(F)** positive nuclear Ki-67 immunoexpression (<5%) (IHC, magnification 200x).

On admission, hemodynamic parameters were stable. General condition was good with a World Health Organization (WHO) performance status score of 0. There was no clinical lymphadenopathy or hepatosplenomegaly. Careful examination of the skin and mucous membranes did not reveal any abnormal or suspicious lesions (Figures 1, 2). A dermoscopy of the giant congenital melanocytic nevus conducted by four independent dermatoscopists did not present any abnormalities. The skin around the giant congenital melanocytic nevus was thin and parchment-like (Figure 3). The rest of the physical and laboratory examination was regular with a normal level of lactate dehydrogenase. In the positron emission tomography (PET) examination, apart from the previously described change in the lower abdomen, no metabolically active foci with features of malignant growth were found. As part of a multidisciplinary consultation, the patient was qualified for surgical removal of the lesion in the lower abdomen. The postoperative histopathological examination confirmed the presence of metastasis of melanoma into the subcutaneous tissue of the abdomen. The nodule of the abdomen was surgically excised with a 1 cm margin. Histopathology of the excised specimen showed tumor-free margins. Microscopic examination revealed a large, expansile nodule with irregular pigmentation, well separated from the epidermis by the rim of normal tissue, and with tumor-free margins. The tumor area was composed of elongated spindle cells (predominantly) and oval epithelioid cells arranged in sheets and bundles. Malignant melanocytes showed atypical, hyperchromatic nuclei and low mitotic count (2–3 mitotic figures per 10 HPF). The cytoplasm was abundant, and a wide range of cells contained a large amount of black pigment granules (Figure 4) Extracellular melanin pigment was also observed. Immunohistochemical staining of the tumor was positive for HMB-45, Melan-A, Vimentin, S-100, SOX-10, and Ki-67 (<5%) (Figure 5), whereas staining for cytokeratin (AE1/AE3) and CD34 was negative. No mutations were detected in proto-oncogene B-Raf (*BRAF*). Based on histopathologic and immunohistochemical examination, the melanoma metastasis was confirmed. The tumor was staged according to the 2018 American Joint Committee on Cancer (AJCC) 8th Edition, and the case was classified as T0N0M1a-IV clinical stage (12). Due to the patient’s RA, the patient was informed about the increased risk of side effects. After a rheumatological consultation and considering the patient’s preferences, she was qualified for adjuvant treatment with nivolumab (240 mg) administered once in 2 weeks for 12 months.

Currently, the patient has completed adjuvant treatment with good tolerance. There was no worsening of rheumatoid arthritis during treatment. The patient remains under the constant care of an oncologist, rheumatologist, and dermatologist.

## 3 Discussion

More than 97% of all melanomas are diagnosed with a known primary origin, mostly involving cutaneous and mucous membranes (13). In rare cases, where a primary lesion is not identified, then it is referred to as a melanoma of unknown primary origin.

Das Gupta et al. were the first to propose the entity MUP in 1963, defining it as melanoma found in subcutaneous tissue, lymph nodes (LNs), or visceral organs without a cutaneous, ocular, or mucosal primary origin (14). The authors also described four exclusion criteria that indicate the recognition of MUP, including no evidence of previous orbital exenteration or enucleation and no evidence of previous skin excision or other surgical manipulation of a mole or birthmark. Lack of inaccurate physical examination, including the absence of an ophthalmologic, anal, and genital examination is also one exclusion criterion for MUP.

Patients with MUP who have nodal disease likely have a similar or better prognosis than patients with stage-matched melanoma of known primary (MKP). However, MUP patients with visceral disease have not been studied as extensively, though it has been shown that they generally have a better prognosis compared to those with disseminated MKP. There is no consensus on the prognostic factors of MUP patients. The management of MUP patients should be the same as those with stage-matched MKP (15). Clinical trials of immunotherapy and targeted therapy in patients with advanced cutaneous melanoma have not explicitly reported response rates specific to MUP patient subgroups due to its low incidence and lack of annotation. However, according to our experience, patients with MUP respond to these new therapies (16).

There are no clear data confirming the benefits of adjuvant therapy in patients with melanoma of unknown primary origin in stage III disease. Patients with an MUP were not eligible for a COMBI-AD clinical trial comparing adjuvant dabrafenib plus trametinib versus placebo in patients with resected, *BRAF*^V600^-mutant melanoma (17). This group of patients was also not included in the analysis of studies assessing the effectiveness of immunotherapy in the adjuvant treatment of patients diagnosed with melanoma at stage III (5, 6). According to the Clinical Study Report for KEYNOTE-054, patients with unknown locations of primary cutaneous melanoma were included in both the pembrolizumab and placebo groups. However, no subgroup analysis of efficacy or safety was carried out for these patients.

In the presented case, it seemed most likely that the primary lesion would be located within the congenital nevus; however, four independent dermatologists conducted a dermoscopic examination of the patient’s skin and mucous membranes. Moreover, four parts of lesions, located within the congenital melanocytic nevi, were qualified for removal. No melanoma was found in any of them. The patient had not previously undergone any other surgical procedures. From birth, she was under the continuous care of a dermatologist and, for the past 5 years, also a rheumatologist. According to the available documentation, the lesion has not changed in size, color, or appearance. Therefore, we could not recognize a melanoma arising in giant congenital melanocytic nevus; however, we should also take into consideration the regression of melanoma area inside giant CMN. At the diagnostic stage of the lesion, we considered clear cell sarcoma, but both the histopathological examination and clinical data, such as the location of the lesion and the age of the patient, suggested melanoma. Unfortunately, we did not have the only tool that could confidently differentiate these lesions in the form of molecular testing EWSR using the FISH method. In addition, the lesion being examined is most likely not a primary lesion. If the potential primary focus was on the skin, it would mean that we did not find it in the clinical examination. Moreover, primary dermal melanoma can be located in the subcutaneous fat and have no connection with the skin, but these cases are extremely rare. Thus, the lack of a primary origin allowed us to conclude melanoma of unknown primary origin with a very high probability.

The pathophysiological mechanism of melanoma of unknown primary origin is not fully understood, and two hypotheses have been proposed. First, the most likely theory of its etiology is an immune-mediated regression of a primary cutaneous origin after metastasis has occurred. The second hypothesis indicates a primary origin from ectopic melanocytes in lymph nodes or viscera (14, 18).

It was revealed that *NRAS* mutation is more frequent in melanomas arising within congenital melanocytic nevi (19). The genetic profiles of different melanomas vary significantly. Many recent studies analyzed the genetic profile of MUP (19–24) and revealed that MUP shares many of the genetic and molecular features of melanoma, which arises in transiently sun-exposed areas of skin. Gos et al. analyzed 102 cases of MUP in patients after therapeutic lymphadenectomy. They revealed that *BRAF* and *NRAS* mutations occurred in 53 and 14% of patients with MUP, respectively. More importantly, *BRAF V600E* mutations comprised 93% of all *BRAF* mutations in that study cohort, and no *c-KIT* mutations were identified, which is more characteristic of melanoma arising from acral skin and mucosal sites (21, 24). In the presented case, the *BRAF V600* mutation was not found in the molecular test. *NRAS* and *c-KIT* mutations were not tested for reimbursement reasons.

Specific guidelines are lacking proper management of melanoma arising in a congenital melanocytic nevus, as well as management of patients with melanoma of unknown primary origin. In both cases, treatment is the same as in the case of patients with metastatic cutaneous melanoma. Surgical excision of lesion or metastasis (if possible, in case of melanoma of unknown primary origin) with adjuvant therapy remains a treatment of choice. In recent years, the management of patients with metastatic melanoma has undergone a revolution because of the introduction of adjuvant systemic treatment based on anti-PD-1-immune checkpoint inhibition therapy (nivolumab and pembrolizumab) or targeted therapy (dabrafenib and trametinib) (25). A choice of adjuvant therapy in the presented cases is guided by studies. Nivolumab was approved by the FDA in December 2017 for adjuvant therapy of cutaneous melanoma with lymph node metastasis and Stage IV disease after resection of metastasis. Other drugs used for adjuvant therapy include pembrolizumab, as well as dabrafenib and trametinib. However, only the study involving nivolumab has evaluated the efficacy of therapy in stage IV melanoma after surgical resection of the metastasis (5–7). We selected nivolumab for drug registration due to its ability to prolong relapse-free survival and its good tolerability. However, its use was controversial given the patient’s existing autoimmune diseases. Due to the patient’s RA, she was informed about the increased risk of side effects related to her joints during immunotherapy. Potential side effects include the possibility of RA exacerbation and the possibility of side effects related to both immunotherapy and methotrexate treatment. After a rheumatological consultation and considering the patient’s preferences, she was qualified for adjuvant treatment with nivolumab. The patient continues treatment with good tolerance. The joint pain did not worsen, and the patient did not require an increase in methotrexate dose.

## 4 Conclusion

Malignant melanoma arising in a giant congenital pigmented nevi is rare and poorly understood. The diagnosis of melanoma metastasis in a patient with a congenital nevus suggests that the primary focus is the nevus. However, as in the presented case, where the primary lesion is not identified, a proper physical examination of the mucous membranes is crucial, and the absence of previous resections of skin lesions does not confirm a diagnosis of melanoma arising from the congenital nevus. Patients with stage IV melanoma of unknown primary origin should be treated aggressively, similar to those with melanoma in the same stage with known origin, with a combination of surgery, immunotherapy, molecularly targeted treatment, and radiotherapy if necessary.

## Data Availability

The original contributions presented in the study are included in the article/supplementary material, further inquiries can be directed to the corresponding authors.
